# *Trypanosoma cruzi* infection follow-up in a sylvatic vector of Chagas disease: Comparing early and late stage nymphs

**DOI:** 10.1371/journal.pntd.0009729

**Published:** 2021-09-20

**Authors:** Valeria Cortés, Amalia Cruz, Sofia Onetti, Daniela Kinzel, Javiera Garcia, Sylvia Ortiz, Angélica Lopez, Pedro E. Cattan, Carezza Botto-Mahan, Aldo Solari

**Affiliations:** 1 Programa Biología Celular y Molecular, ICBM, Facultad de Medicina, Universidad de Chile, Santiago, Chile; 2 Facultad de Ciencias Veterinarias y Pecuarias, Universidad de Chile, Santiago, Chile; 3 Facultad de Ciencias, Universidad de Chile, Santiago, Chile; Universidade Federal de Minas Gerais, BRAZIL

## Abstract

Chagas disease is caused by *Trypanosoma cruzi* and transmitted by the triatomine *Mepraia spinolai* in the southwest of South America. Here, we examined the *T*. *cruzi*-infection dynamics of field-caught *M*. *spinolai* after laboratory feeding, with a follow-up procedure on bug populations collected in winter and spring of 2017 and 2018. Bugs were analyzed twice to evaluate *T*. *cruzi-*infection by PCR assays of urine/fecal samples, the first evaluation right after collection and the second 40 days after the first feeding. We detected bugs with: the first sample positive and second negative (+/-), the first sample negative and second positive (-/+), and with both samples positive or negative (+/+; -/-). Bugs that resulted positive on both occasions were the most frequent, with the exception of those collected in winter 2018. Infection rate in spring was higher than winter only in 2018. Early and late stage nymphs presented similar *T*. *cruzi*-infection rates except for winter 2017; therefore, all nymphs may contribute to *T*. *cruzi*-transmission to humans. Assessment of infection using two samples represents a realistic way to determine the infection a triatomine can harbor. The underlying mechanism may be that some bugs do not excrete parasites unless they are fed and maintained for some time under environmentally controlled conditions before releasing *T*. *cruzi*, which persists in the vector hindgut. We suggest that *T*. *cruzi*-infection dynamics regarding the three types of positive-PCR results detected by follow-up represent: residual *T*. *cruzi* in the rectal lumen (+/-), colonization of parasites attached to the rectal wall (-/+), and presence of both kinds of flagellates in the hindgut of triatomines (+/+). We suggest residual *T*. *cruzi*-infections are released after feeding, and result 60–90 days after infection persisting in the rectal lumen after a fasting event, a phenomenon that might vary between contrasting seasons and years.

## Author summary

In the vector-borne transmission of Chagas disease, approximately 150 species of triatomine bugs are potential vectors for the parasite *Trypanosoma cruzi*. A competent vector must fulfill several features such as the ability to host, amplify, and differentiate the parasite, allowing *T*. *cruzi* persistence within the insect vector. Our aim was to describe the dynamics of *T*. *cruzi* infection in a competent triatomine species collected in two contrasting seasons—with different environmental temperatures—of 2017 and 2018. We used a follow-up procedure including *T*. *cruzi* detection right after collection and 40 days later; both detections were performed after laboratory feeding. Most infected bugs were *T*. *cruzi* positive on both occasions. However, infected bugs from winter 2018 presented switches from *T*. *cruzi* negative at collection to *T*. *cruzi* positive 40 days later. The results suggest infections with *T*. *cruzi* attached to the hindgut wall as the colonization site, caused by infections that persist there after a fasting event, are released after a second feeding.

## Introduction

Climatic variation is a major determinant of infectious disease dynamics [1]. Insects are ectotherms with a relatively small range of temperatures that can be endured, and in which activity takes place, with variable low thermal limits and narrow upper thermal tolerance [2]. Pathogen transmission in vector-borne diseases can be measured by the basic reproductive number. This epidemiological metric depends on vector and parasite traits including vector competence and density, which can be partitioned as the product of several parameters including development rate, vector survival, feeding, dispersal and reproduction, all dependent on temperature and affecting population abundance [3,4].

Chagas disease, caused by the protozoan parasite *Trypanosoma cruzi*, is the main neglected vector-borne disease in America [5]. This disease presents at least two phases, acute and chronic. The agent, *T*. *cruzi*, is a stercorarian trypanosome transmitted by hematophagous long-lived insect vectors of the subfamily Triatominae. The infection begins when the fecal/urine excretion of an infected triatomine contacts the host in the skin wounds, mucous membranes, or by oral ingestion. Thus vector competence depends on several factors including the parasite infection prevalence and the parasite burden in different seasons of the year. As shown in early studies, the highest number of acute cases of Chagas disease occurs during hot seasons; therefore disease occurrence has been associated with temperature changes in different seasons in southern latitudes [6–8], since seasonal temperature variation increases with latitude [9]. Even though some information has been reported on the effect of temperature on domestic triatomine species, little is known about its effect on infected sylvatic triatomine species with a relevant role in *T*. *cruzi* maintenance and interplay between the domestic and sylvatic transmission cycles of Chagas disease.

*T*. *cruzi* is a unicellular flagellate trypanosomatid, with a single mitochondrion containing kinetoplast DNA composed of concatenated maxicircles and minicircles [10]. Due to the high number of minicircles, these are appropriate targets for PCR DNA-based detection of *T*. *cruzi*. Both epimastigotes (non-infective forms) and trypomastigotes attach to the cuticular lining and colonize the rectum [11]. *T*. *cruzi* competent vectors quickly urinate/defecate several drops after a blood meal. *T*. *cruzi* attaches to the cuticle of the rectum, covered by a wax layer over the external epicuticle [12]. The parasite amplifies as epimastigotes in the posterior midgut and hindgut, increasing the parasite density there after infection [13–15]. Later on, triatomines release metacyclic trypomastigotes (infective forms) after metacyclogenesis of the epimastigotes attached to the cuticular lining, another important aspect to determine vector competence [16]. After an extensive blood meal, the triatomine abdominal distension induces a rapid diuresis, and subsequently molting and ecdysis several days later. Parasite density in the hindgut is negatively influenced by fasting [13,17,18], or by regular feeding without reinfection [19–21].

At least three endemic secondary and sylvatic triatomine species are present in the subtropical Pacific side of South America, specifically north-central Chile (~18°S to 34°S), which have arid and semiarid-Mediterranean climatic areas. The triatomine *Mepraia spinolai* [22] mainly inhabits interior valleys up to 3000 m elevation (26°-34° S), where it feeds on mammal species [23–25], with two rodent species (*Phyllotis darwini* and *Octodon degus*) as the most frequent blood sources [24,26]. However, several home as well as peridomiciliary invasion complaints are notified each year [27,28], with human blood as part of the diet [24,25]. Even though vector-borne transmission by the domestic vector *Triatoma infestans* was declared interrupted in Chile in 1999, wild vectors are still an important problem in endemic areas [29,30] and their study should be a priority in public health programs.

*M*. *spinolai* is distributed in areas with highly variable minimum and maximum temperatures depending on the season of the year, exhibiting an aggregated distribution using rocky outcrops and bromeliads as refuges, where they coexist with small wild mammals [31–33]. This triatomine bug has reduced dispersal capability, with higher activity during the photophase and larger home range in summer than winter [34,35]. In warm seasons, *M*. *spinolai* takes blood meals on a wider range of hosts compared to the fall season, suggesting that feeding is associated with a greater dispersion pattern and greater host availability [24,35]. Populations of *M*. *spinolai* collected in warm seasons present highly variable *T*. *cruzi* infection rates [31,32,36,37], in which the rodent *P*. *darwini* accounts for most variation in vector infection risk [33].

*M*. *spinolai* may reach very high densities and exhibits variable population structure depending on the season, with a high proportion of I to III stage nymphs in late austral summer-fall (January to April) and all development stages in austral spring-early summer (September to December) [38–40]. A recent regional study (26°-31°S) showed that mean temperature of the warmest trimester is positively associated with *M*. *spinolai* abundance and *T*. *cruzi* infection [32]. A small spatial scale study showed that *T*. *cruzi* infection in *M*. *spinolai* is higher in spring and summer compared to fall, and survivorship of the second stage nymphs is lower in spring than in the other seasons [41].

Competent triatomine vectors may harbor *T*. *cruzi* for long time, an epidemiological trait associated with the risk of transmission to humans, but despite its relevance, there is no reported information about the dynamics of this harboring process in different stage nymphs of sylvatic triatomines captured during contrasting seasons. In this study we used field-caught *M*. *spinolai* collected during the austral winter and spring seasons of 2017 and 2018 to answer the following questions: (i) Does *T*. *cruzi* infection prevalence in *M*. *spinolai* vary between two contrasting seasons of two consecutive years? (ii) Do naturally infected *M*. *spinolai*, fed in laboratory conditions, provide relevant information to assess variation in *T*. *cruzi*-infection dynamics? (iii) Does vector development stage (early and late nymphs) associate with *T*. *cruzi* infection prevalence depending on the follow-up and season?

## Methods

### Ethics statement

Mice (age 2 months) were obtained from the vivarium facilities of the Faculty of Medicine, University of Chile. All procedures of animal handling carried out in this study were performed according to the rules and with the permission of the Animal Ethics Committee of the University of Chile (CBA#0443-FMUCH-2011; CBA#0987-FMUCH-2018).

### Triatomine collection and temperature data

**Fig 1:**
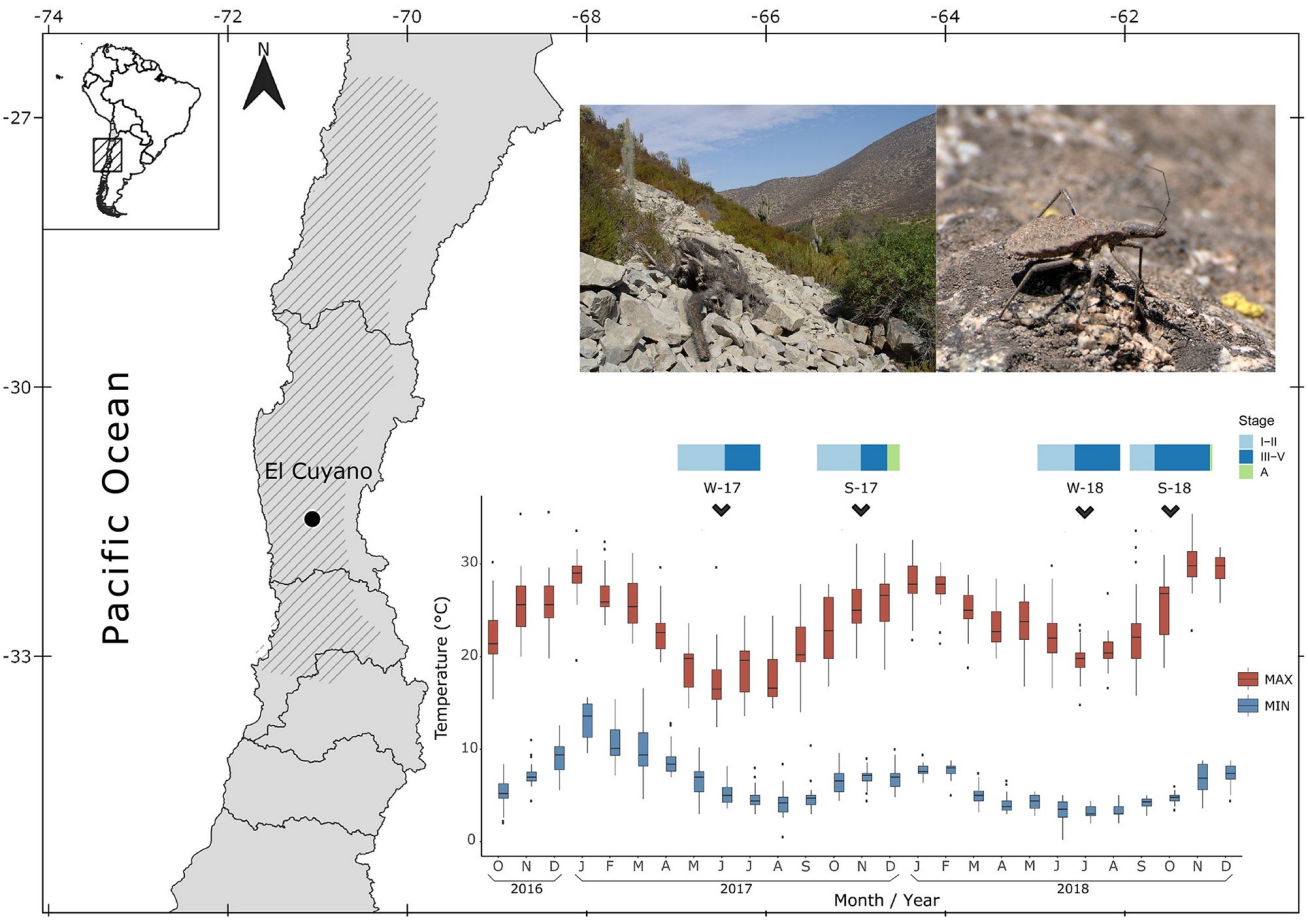
Study site and environmental temperatures. Map of North-central Chile showing in hatch pattern the distribution of *Mepraia spinolai* and the study site, El Cuyano (left). Photographs of the rocky outcrop where *M*. *spinolai* are collected and a late stage nymph of *M*. *spinolai* (top right). Boxplot of minimum (blue boxes) and maximum (red boxes) temperatures from October 2016 to December 2018, indicating the capturing month of the four groups of *M*. *spinolai* under study: winter and spring 2017 (W-17, S-17) and 2018 (W-18, S-18) (bottom right). The proportion of collected bugs per development stage is shown for each capture event: early (I and II) and late (III, IV, and V) stage nymphs, and adults (A). Photos by Carezza Botto-Mahan. Base layer of the map available at https://www.ide.cl/index.php/informacion-territorial/descargar-informacion-territorial.

The field work was carried out at El Cuyano (31°29’01”S, 71°03’40” W; Coquimbo Region, Chile; Fig 1), a locality included in a hyperendemic area of Chagas disease, during the winter and spring seasons of 2017 and 2018. Minimum and maximum temperatures were obtained from the weather station of Las Chinchillas National Reserve (National Forest Corporation, Ministry of Agriculture of Chile; Supporting information S1 Table. Environmental temperature data), a protected area located ~5.9 km distant from the *M*. *spinolai* collection site. The study site has a semiarid-Mediterranean climate with widely variable temperatures throughout the year, scarce plant cover dominated by bromeliads, large areas of rock piles, pebbles and sand, and with several domestic (goats, sheep, dogs), native and free-ranging introduced small mammals.

Triatomine bugs were collected manually as bugs approached in an area of ~1680 m^2^ from 11:00 to 16:00 by a trained researcher acting as human bait. Bugs were collected during four and two consecutive days in the winter and spring seasons, respectively. Collected bugs were stored in separate containers with folded paper as refuge and transported in polystyrene foam boxes to the laboratory. Information on each *M*. *spinolai* collection event is provided in Table 1. A small fraction of nymphs did not resist the trip and arrived dead to the laboratory, mostly starved first-stage nymphs that probably had never fed on a host. These nymphs were not included in the analyses.

**Table 1:** Number of *Mepraia spinolai* individuals, shown by development stage, collected in the field in two seasons of two consecutive years and laboratory fed.

Group code	Season/year	Stage	Total	Number of bugs		Dead
				Molted	Non-molted	
W-17	Winter 2017	I-II	42	37	3	2
		III-IV-V	32	16	15	1
		Adults	0	-	-	-
S-17	Spring 2017	I-II	30	19	11	0
		III-IV-V	18	6	11	1
		Adults	9	-	-	0
W-18	Winter 2018	I-II	61	47	11	3
		III-IV-V	76	59	17	0
		Adults	0	-	-	-
S-18	Spring 2018	I-II	60	34	26	0
		III-IV-V	134	51	82	1
		Adults	5	-	-	0

Number of *M*. *spinolai* molted, non-molted and dead after one feeding event under laboratory conditions. I-II: 1^st^ and 2^nd^ stage nymphs (early stage nymphs); III-IV-V: 3^rd^, 4^th^ and 5^th^ stage nymphs, respectively (late stage nymphs).

### Triatomine fecal sample collection after laboratory feeding

Bugs arrived at the laboratory 1–2 days after collection, where they were classified by development stage and individually housed in a plastic box with several small-size compartments (3.2 × 3.6 × 1.5 cm) with an identification tag. All bugs were maintained in a growth chamber under optimal conditions at 26 °C, 75% relative humidity and 14:10 (L:D) h cycle [41]. Over a one week period after arrival, bugs were individually fed on uninfected mice (*Mus musculus*) anesthetized with 2% sodium thiopental.

To avoid bug harm and to allow further follow-up evaluation, fecal samples were obtained after spontaneous defecation up to 30 min after feeding, which were mixed with 100 μL of distilled water for molecular analyses. In addition, one small volume of each fresh fecal sample was diluted 10 times in saline buffer to be examined microscopically, by searching for *T*. *cruzi* (free swimming and non-motile flagellates) during 10 min under 100× magnification, following the method described for blood samples [42] adapted for fecal samples. The first feeding nourished the bugs and kept them alive until the second collection of feces/urine samples repeated 40 days later, following the same feeding procedure. Molting and survival were recorded weekly after the first feeding. Dead bugs after the first feeding event were recorded but not included in statistical analyses.

### DNA extraction and detection of *T*. *cruzi* infection

Each fecal sample was extracted in a final volume of 200 μL with conditions already described with the EZNA Blood DNA Mini kit (OMEGA BIO-TEK, Norcross, GA, USA). *T*. *cruzi* infection was determined three times by PCR directed to minicircle kDNA with oligos 121 and 122 using different volumes of extracted DNA as template (range of 2–6 μL) in a final volume of 50 μL [43]. A 10 μL volume of each reaction mixture was run in an agarose gel electrophoresis and stained with ethidium bromide. PCR assays were repeated when negative results were obtained. In those cases, the extracted DNA was concentrated three times by evaporation. Three different PCR assays were repeated using variable volumes of the concentrated DNA as template. A negative sample resulted when all PCR attempts with extracted DNA failed to detect a 330 bp amplicon.

### Statistical analyses

We tested for differences in minimum and maximum temperatures between the same austral seasons in the two sampled years by Kruskal-Wallis tests using two months in each season. The association between follow-up/season/development stage and *T*. *cruzi* prevalence was tested by χ^2^ tests for: (i) follow-up within each season and year, (ii) seasons within year (winter/spring) considering follow-up, and (iii) development stage within each season and year. The significance level (or alpha level) considered statistically significant in this study was 0.05. Analyses were performed with R (version 3.6.0, R Development Core Team 2019) or JMP-Pro (version 14).

## Results

### *M*. *spinolai* collection and environmental temperatures

Table 1 shows the distribution of nymphs and adults collected in the austral winter and spring of 2017 (groups W-17 and S-17, hereafter) and 2018 (groups W-18 and S-18, hereafter), shown as number of first and second stage nymphs (early stage nymphs, hereafter), and third, fourth and fifth stage nymphs (late stage nymphs, hereafter). This table also shows the number of bugs that molted after the first feeding and died during the follow-up. Only 14 out of 467 bugs were adults, collected exclusively in spring seasons (S-17 and S-18). Minimum and maximum temperatures revealed statistically significant differences between the studied years (Fig 1). The maximum temperature increased almost 5 °C in winter 2018 compared to winter 2017 (max. median T°: W-17 = 15.6 °C, W-18 = 20.4 °C; χ^2^ = 40.01, *p* < 0.0001), and 3.4 °C in spring 2018 compared to spring 2017 (max. median T°: S-17 = 24.4 °C, S-18 = 27.8 °C; χ^2^ = 29.65, *p* < 0.001). The minimum temperature decreased almost 1°C during winter 2018 compared to winter 2017 (min. median T°: W-17 = 4.0 °C, W-18 = 3.2 °C; χ^2^ = 39.75, *p* < 0.001), and 1.8 °C in spring 2018 compared to spring 2017 (min. median T°: S-17 = 6.8 °C, S-18 = 5.0 °C; χ^2^ = 15.98, *p* < 0.001). Because of the statistical differences in minimum and maximum temperatures between the same seasons of the years, data of different years were not combined for posterior analyses. Complete environmental temperature data in S1 Table.

### *T*. *cruzi* infection dynamics and microscopic observation

**Fig 2:**
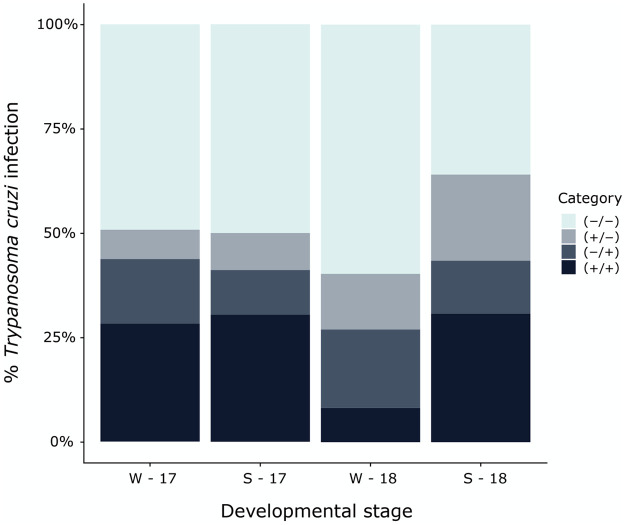
Percentage of *Mepraia spinolai* (all development stages combined) with presence of *Trypanosoma cruzi* DNA detected by PCR in fecal/urine samples during two laboratory feedings, in the four collecting events (W-17, S-17, W-18, S-18). The four types of PCR results are shown: both fecal samples positive (+/+), first fecal sample negative and second positive (-/+), first fecal sample positive and second negative (+/-), and both fecal samples negatives (-/-).

Table 2 shows the results of PCR assays of samples obtained with the first fecal/urine sample (i.e., bugs analyzed upon arrival) and those with the second fecal/urine sample (surviving bugs analyzed 40 days after the first laboratory feeding). Three kinds of PCR positive results were recorded: (i) bugs with the first fecal sample positive and the second negative ((+/-), hereafter), (ii) bugs with the first fecal sample negative and the second positive ((-/+), hereafter), and (iii) bugs with both fecal samples positive ((+/+), hereafter) (S1 Fig shows PCR results of DNA bands from representative samples of *T*. *cruzi* infected and uninfected bugs during the follow-up). The number of cases (all development stages combined) of each of the four potential results (+/+, +/-, -/+, -/-) depicted in Table 2 are shown in Fig 2 as percentages for each *M*. *spinolai* group.

**Table 2:** PCR results and percentage of *Trypanosoma cruzi* infection in *Mepraia spinolai* collected in winter (W) and spring (S) of 2017 (17) and 2018 (18) surviving two feeding events, shown combined and by early (I-II) and late (III to adults) stages.

Group code	Development stage	N° bugs tested	N° bugs (first PCR/second PCR)				Non-infected (%)	Infected (%)	
			(+/-)	(+/+)	(-/+)	(-/-)	(-/-)	First PCR	Follow-up
W-17	All	71	5	20	11	35	49.3	28.2	43.7
	I-II	40	3	4	2	31	77.5	10.0	15.0
	III to V	31	2	16	9	4	12.9	51.6	80.7
S-17	All	56	5	17	6	28	50.0	30.4	41.1
	I-II	30	3	7	1	19	63.3	23.3	26.7
	III to Adults	26	2	10	5	9	34.6	38.5	57.7
W-18	All	134	18	11	25	80	59.7	8.2	26.9
	I-II	58	7	5	14	32	55.2	8.6	32.8
	III to V	76	11	6	11	48	63.2	7.9	22.4
S-18	All	198	41	61	25	71	35.9	30.8	43.4
	I-II	60	11	24	7	18	30.0	40.0	51.7
	III to Adults	138	30	37	18	53	38.4	26.8	39.9

First PCR performed upon arrival at the laboratory and the second 40 days later. (+/-): first positive and second negative; (+/+): both positive; (-/+): first negative and second positive; (-/-): both negative. First PCR includes (+/+) only; follow-up includes (+/+) and (-/+). (+/-) probably corresponds to bugs that excreted dead parasites in the first PCR (i.e., with *T*. *cruzi* DNA remains).

Overall, 38.3% of the tested bugs were *T*. *cruzi* infected in the follow-up, but only 25.1% showed evidence of infection (mostly metacyclic trypomastigotes and few non-motile flagellates) by means of microscopy including results after the two feeding events combined (W-17: 28.2%, S-17: 32.1%, W-18: 14.9%, S-18: 28.8%; Supporting information S1 Table). All groups, with the exception of W-18, exhibited a higher proportion of bugs (all development stages combined) that maintained *T*. *cruzi* infection (+/+) (W-17: χ^2^ = 14.25, *p* < 0.001; S-17: χ^2^ = 14.25, *p* < 0.001; S-18: χ^2^ = 23.05, *p* < 0.001). In contrast, in W-18 switching from (-) to (+) was the most frequent type of infection (χ^2^ = 8.17, *p* = 0.017). No significant differences were detected between seasons for either years in the proportion of bugs (all development stages combined) exhibiting (+/-) results (W-17 vs S-17: χ^2^ = 0.19, *p* = 0.665; W-18 vs S-18: χ^2^ = 0.019, *p* = 0.890).

### Effect of follow-up on *T*. *cruzi* detection

**Fig 3:**
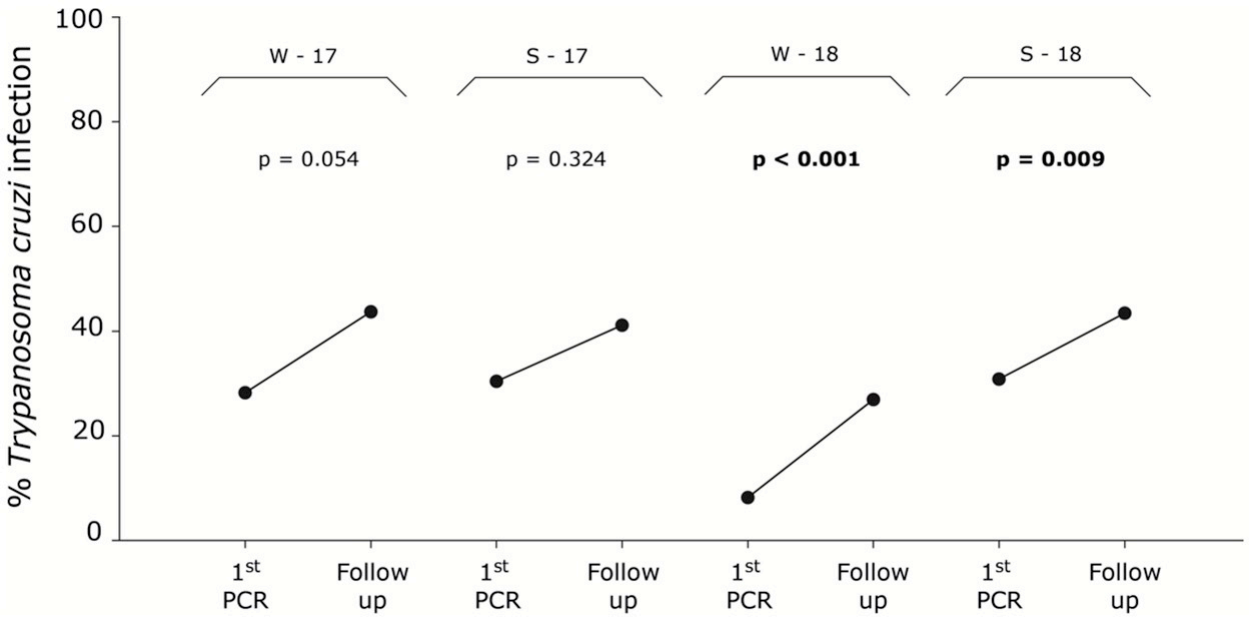
Percentage of *Trypanosoma cruzi* infection (all development stages combined) in each *Mepraia spinolai* group (W-17, S-17, W-18, S-18) for the first PCR (+/+) only or follow-up (+/+ and -/+). P-values are shown above each comparison. Statistically significant differences are in bold. (+/-) probably corresponds to bugs that excreted dead parasites in the first PCR (i.e., with *T*. *cruzi* DNA remains).

Because +/- results probably correspond to bugs that excreted dead parasites in the first PCR (i.e., with *T*. *cruzi* DNA remains), they were not considered as *T*. *cruzi* infected bugs in all posterior statistical analyses. Comparisons of *T*. *cruzi* prevalence (all development stages combined), for each group and year between the first PCR only or both PCR (follow-up) revealed that the follow-up detected significantly more *T*. *cruzi* infection in some of the tested groups (Table 2 and Fig 3). Specifically, in winter groups the follow-up detected marginally (W-17: χ^2^ = 3.70, *p* = 0.054) and significantly (W-18: χ^2^ = 16.13, *p* < 0.001) more infection. The follow-up detected significantly more infection only in spring 2018 (S-18: χ^2^ = 6.76, *p* = 0.009). The follow-up did not detect more *T*. *cruzi* infection in early stage nymphs, except for those nymphs collected in W-2018 (χ^2^ = 10.30, *p* = 0.001). In late stage nymphs, the follow-up showed almost the same pattern as for all development stages combined (W-17: χ^2^ = 5.83, *p* = 0.016; W-18: χ^2^ = 6.20, *p* = 0.013; S-18: χ^2^ = 5.28, *p* = 0.022).

### Effect of season on *T*. *cruzi* infection prevalence

Comparison of *T*. *cruzi* prevalence (all development stages combined) between seasons of the same year, with the first PCR only or both PCR (follow-up), revealed significant differences for 2018 only. Specifically, in spring 2018 we detected more *T*. *cruzi* infection prevalence than winter, considering both the first PCR only (χ^2^ = 24.03, *p* < 0.001) and the follow-up (χ^2^ = 9.44, *p* = 0.002). The same pattern as for all development stages combined was detected in early and late stage nymphs in 2018. Conversely, in 2017 we detected marginally significant difference between seasons only in late stage nymphs with the follow-up procedure (χ^2^ = 3.56, *p* = 0.059).

### Effect of development stage on *T*. *cruzi* infection prevalence

*T*. *cruzi* prevalence between early and late stage nymphs within the same season and year, considering the first PCR only, revealed differences in only one group. Specifically, higher *T*. *cruzi* prevalence was detected in W-17 in late stage nymphs compared to early ones (χ^2^ = 14.95, *p* < 0.001). No statistically significant differences were detected in the other groups (S-17, W-18, and S-18).

## Discussion

We performed a *T*. *cruzi* follow-up in naturally infected bugs subjected to laboratory feedings, one feeding right after collection and a second 40 days later. Overall, in spring we detected more *M*. *spinolai* individuals infected than in winter, both right after collection and after feeding in the lab (follow-up). The same pattern was reported by a previous study carried out in one location nearby our study site [31], in which summer infection was higher than that detected in winter bugs right after capture. However, unlike previous studies, the follow-up procedure would allow to find out the type of infection a bug is experiencing when captured under field conditions, rendering valuable information on vector infectivity.

In our study, the most frequent infection corresponded to bugs that excreted *T*. *cruzi* after the first and the second feeding (+/+), which represents an active infection, i.e., bugs with parasites in the rectal lumen as well as attached to the hindgut wall. Other individuals switched from positive to negative PCR results (+/-), which could represent a residual infection with *T*. *cruzi* only in the rectal lumen of starved insects, probably occurring 60–90 days after infection, with dead detached parasites as previously reported [17]. However, other factors such as the insect immune response might explain this result. The infection (-/+) could represent insects with *T*. *cruzi* attached to the hindgut. This situation probably occurs in more recent infections, when parasites attached to the hindgut are released after feeding and the excreta analyzed 40 days later or less [17]. Nonetheless, only insect dissection might allow to evaluate where *T*. *cruzi* is developing.

Early and late stage nymphs (including adults) from both seasons and years were analyzed all together and separately to gather information on development stage-dependent *T*. *cruzi* prevalence and infection risk, especially because nymphs of different development stages might present contrasting eco-physiological traits (e.g., dispersal capability, temperature endurance) [44]. Higher *T*. *cruzi* prevalence with the follow-up procedure was detected in late stage nymphs in three out of the four studied groups (except for spring 2017) compared to the prevalence detected with the first PCR only. In early stage nymphs the follow-up procedure was able to detect higher *T*. *cruzi* prevalence only in winter 2018. This last result probably corresponds to newly infected early stage nymphs, after feeding on infected mammals in an unusual warmer winter such as that of 2018, especially if we consider that *M*. *spinolai* is active over 15 °C [34,35]. In summary, we suggest that the results of the first PCR would be showing vector infectivity (at the time of capture), but the follow-up would be detecting also ongoing infection within a vector [21].

*T*. *cruzi* prevalence was higher in early stage nymphs in spring than in winter of 2018 only, using both detection procedures. In general, late stage nymphs from both spring seasons presented higher *T*. *cruzi* prevalence than those from winter seasons. We suggest that minimum and maximum environmental temperatures perceived by the winter and spring 2018 groups were more contrasting than those of 2017 groups. Even though we detected some interannual variation, we suggest that environmental factors translated into higher *T*. *cruzi* prevalence in the warmer season of 2018, indicating that season could be a relevant factor when assessing infection risk in austral latitudes. Metacyclogenesis is a temperature-dependent process [45]. For example, at 22–23 °C fewer metacyclic trypomastigotes are excreted by triatomines, but many more are released at 26–30 °C [45,46]. In other study, trypomastigotes appeared in the rectum and feces earlier at 28° than at 20°C, but after that time triatomines developed similar population densities [47], probably reaching their carrying capacities. We found mostly metacyclic trypomastigote forms by optical microscopy in the excreta of highly infected *M*. *spinolai* in spring, supporting that *T*. *cruzi* infective forms are released with high infectivity to different hosts mostly in warmer seasons. The sylvatic species *Triatoma brasiliensis* presented higher *T*. *cruzi* infection rates in equator latitudes of Brazil in the rainy season than in the dry season [48]. These results indicate that in areas with subtle changes in environmental temperatures, other variables such as rainfall rather than temperature may determine vector infectivity.

Late stage nymphs resulted in higher *T*. *cruzi* infections determined with the first PCR assay than early stage nymphs collected in winter 2017, but not in equivalent bugs of winter 2018. Late stage nymphs of winter 2017 were probably more resistant to low temperatures and may have dispersed larger distances to search for a host than early stage nymphs, having more opportunities to survive and become infected by *T*. *cruzi*.

The different infection detected between early and late stage nymphs of the two studied winters suggests that subtle changes in environmental threshold temperatures might determine when *M*. *spinolai* enter fasting, therefore reducing the probability of infection or re-infection. Contrarily, in both springs, similar *T*. *cruzi* prevalence was detected in early and late stage nymphs. In consequence, both types of nymph stages might be equally risky for mammals including humans in transmitting *T*. *cruzi* in warmer seasons and, probably, in unusually warm winters.

During the follow-up we detected +/- residual infections for bugs that excrete the last fraction of probably dead parasites from the rectal lumen after a long fasting event. We found that this kind of result was not associated with the studied seasons, because not only winter bugs presented residual infection (+/-), but also spring bugs, suggesting that fasting also occurs in nature in temperate weather.

Different extensions of fasting up to starvation affect the nutritional status of triatomines altering host detection and approaching behavior [49]. A field study detected that *T*. *cruzi*-infected *M*. *spinolai* with the highest nutritional status approached humans first [40]. Those results along with the present study in *M*. *spinolai* of different seasons are epidemiologically important to determine the *T*. *cruzi* infectivity risk for humans in warm seasons. The two kinds of residual infection detected with a follow-up have a timing not yet determined. However, in *T*. *infestans* experimental infection with *T*. *cruzi* (strain Chile 5) under fasting, an old infection is close to 30 days and a much older infection can last 60 or more days with an extensive killing of flagellates in the rectum [17,50]. A long fast in *M*. *spinolai* during winter can last 60–90 days, probably without molting, a period of time this species can withstand. Under optimal laboratory conditions, uninfected *M*. *spinolai* individuals subjected to 100 days of fasting survived more than those *T*. *cruzi*-infected *M*. *spinolai* [18]. *M*. *spinolai* of different development stages submitted to fasting after full engorgement had life spans of 70–300 days [41], suggesting that *M*. *spinolai* can withstand long periods of fasting like those it undergoes in winter.

In conclusion, all development stages may contribute equally to *T*. *cruzi* transmission to humans mainly, but not exclusively, in warmer seasons. Future studies, using more sensitive detection methods, should focus on parasitic burden follow-up in sylvatic triatomines and the interannual variation in *T*. *cruzi* prevalence to assess the complete scenario of transmission in austral latitudes of South America.

## Supporting information

S1 Table Microscopy observation and PCR results for *M*. *spinolai*, and environmental temperature data. Detailed information for each *M*. *spinolai* individual indicating season and year, development stage, first and second optical microscopy and PCR results, and molting. Minimum and maximum daily temperatures from October 2016 to December 2018 from the National Forest Corporation (CONAF) weather station located at Las Chinchillas National Reserve, Coquimbo Region, Chile.
(XLSX)

S1 Fig Representative results of PCR assays on fecal/urine samples of some *M*. *spinolai*. (TIFF)
