# Supplementary figures and images for: Trypanosoma cruzi infection follow-up in a sylvatic vector of Chagas disease: Comparing early and late stage nymphs
Source: PLoS Negl Trop Dis. 2021 Sep 20;15(9):e0009729. doi: 10.1371/journal.pntd.0009729 (PMC8452000; doi:10.1371/journal.pntd.0009729)

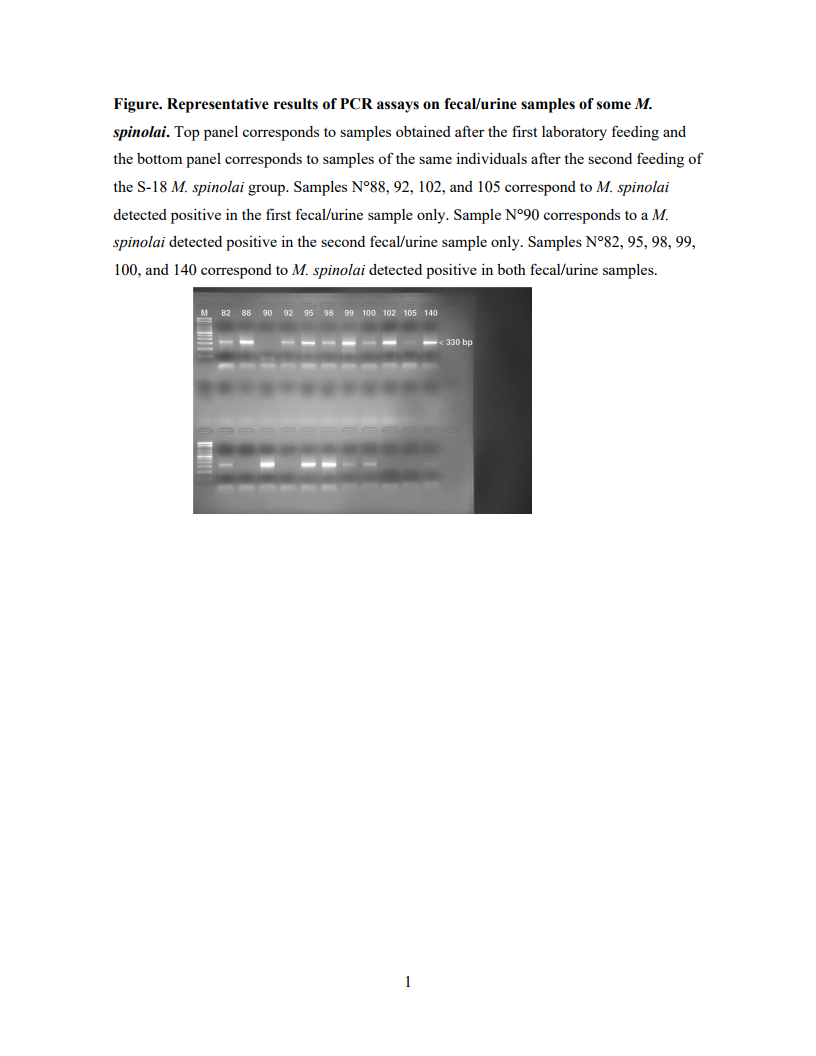

Supplement: S1 Fig — (TIFF) [file pntd.0009729.s002.tiff]
